# Sex effects on early life features of the gingival transcriptome

**DOI:** 10.3389/fdmed.2025.1653315

**Published:** 2025-10-28

**Authors:** J. L. Ebersole, S. S. Kirakodu, L. M. Nguyen, O. A. Gonzalez

**Affiliations:** ^1^ Department of Biomedical Sciences, School of Dental Medicine, University of Nevada Las Vegas, Las Vegas, NV, United States; ^2^ Center for Oral Health Research, College of Dentistry, University of Kentucky, Lexington, KY, United States

**Keywords:** nonhuman primate, aging, sex, transcriptome, periodontitis

## Abstract

**Background:**

Epidemiologic assessment of periodontitis prevalence and extent demonstrates age, sex, and race/ethnicity effects. However, the biological sources of these observations regarding sex differences with an elevated incidence in males remain unclear.

**Methods:**

This study used a model of experimental ligature-induced periodontitis in young nonhuman primates (*Macaca mulatta*) to evaluate gingival transcriptomic differences stratified based on the sex of the animal. The animals represent humans aged 10–25 years of age, with gingival tissue samples obtained at baseline, 0.5 months (initiation), and 1 and 3 months (progression). Microarray analysis was used to quantify gene expression profiles in the gingival tissues.

**Results:**

The results demonstrated clear gene expression differences in healthy (baseline) tissues between the sexes, with elevations in females associated with immune responses and elevated gene expression in males related to tissue structural genes. With disease initiation, fewer genes differed between the sexes, although a pattern of a greater number of unique gene expression changes was observed in females at late progression. Overexpressed biological processes showed tissue structural/functional genes at initiation, with host response pathways altered during disease progression.

**Conclusion:**

These findings support that this model can be used to explore processes that contribute to sex as a biological variable in periodontitis.

## Introduction

Periodontitis represents an emergence of dysbiotic microbial biofilms in the subgingival sulcus that elicit a persistent inflammatory response, creating the hallmarks of this chronic disease (1). The dysbiosis creates more pathogenic biofilms (2) and result in altered innate immune, inflammatory, and adaptive immune response cells and biomolecules in the lesions (3–8). Clear variations in response profiles at the individual patient level are observed related to features of the microbial biofilm stimulus, behavioral/environmental perturbations, and genetic control of host responses (9). Epidemiologic analyses have demonstrated significant variations in disease extent/severity related to age (10, 11), race/ethnicity (12, 13), sex (14, 15), health behaviors (16, 17), and systemic conditions (18, 19). However, a conundrum of this disease is that the oral bacteria with the potential to help drive the disease process and the capacity of the inflammatory/immune system exist early in life, but the disease is generally not observed in humans until after 3 decades of life (20). Thus, there remains a gap in knowledge regarding if there are early events in the mucosal gingival tissues that can predispose to a greater likelihood of periodontitis later in life.

Nonhuman primates have been used for over 50 years to model naturally-occurring periodontitis lesions (21, 22), as well as providing the capacity to initiate experimental periodontitis via ligating teeth (23). Features of this model have shown that periodontitis prevalence is increased with age (24), in males (25), impacted by obesity (26, 27), and demonstrates heritability of periodontitis risk in rhesus monkeys (28) similar to data from human populations. Previous studies have also shown variations in oral microbomes in health and disease (29, 30) based upon the age of the animals. Local/systemic inflammatory and immune response biomolecules based upon the sex of the animal have been reported (25), as well as substantial differences in the gingival transcriptome of an adult population of the nonhuman primates stratified based upon sex of the animals (23). This report extends those findings to describe sex effects on the gingival mucosal tissue transcriptome in young animals in health and disease. The focus was to address features of the gingival tissues in younger individuals, where sex as a biological variable was considered. These findings may be able to contribute to the identification of targeted analytes for early recognition of disease risk, as well as the potential to detect biological differences enabling sex-specific interventions.

## Methods

### Nonhuman primate model and oral clinical evaluation

Rhesus monkeys (*Macaca mulatta*) were housed at the Caribbean Primate Research Center (CPRC) at Sabana Seca, Puerto Rico, for this study. Healthy animals were <3–7 years of age, [∼10–25 human years; (*n* = 18; 9 females and 9 males)] representing a convenience sample based upon availability and cost of the experimental procedures. The animals are typically fed a 20% protein, 5% fat, and 10% fiber commercial monkey diet (diet 8,773, Teklad NIB primate diet modified: Harlan Teklad), supplemented with fruits and vegetables, and water is provided *ad libitum* in an enclosed corral setting.

A protocol approved by the Institutional Animal Care and Use Committee (IACUC) of the University of Puerto Rico enabled anesthetized animals to be examined for clinical periodontal measures, including probing pocket depth (PPD), and bleeding on probing (BOP), as we have described previously (24). The animals were be sedated with Ketamine (10–22 mg/kg) alone or dexmedetomidine (0.02–0.03 mg/kg) using a 3 ml syringe with a 1.5 inch, 22 gauge needle. A full mouth examination, excluding 3rd molars and canines, by a single investigator (Dr. Luis Orraca) using a Maryland probe on the facial aspect of the teeth, e.g., 2 interproximal sites *per* tooth (mesio- and disto-buccal) was conducted. Eighteen periodontally healthy animals classified by a mean BOP of ≤1.0 and a mean PPD of ≤3.0 were included in a standard experimental ligature-induced periodontitis study (31, 32), while naturally-occurring periodontitis animals with a mouth mean BOP index of ≥2.0 and a mouth mean PPD of >3.0 were excluded from the study. Baseline (healthy) gingival tissues were biopsied from each animal from a single site before the experimental periodontitis procedure. Ligature-induced periodontal disease was initiated whereby 2nd premolar and 1st and 2nd molar teeth in all 4 quadrants were ligated by tying 3-0 non-resorbable silk sutures around the cementoenamel junction of each tooth and using a periodontal probe to position the ligatures below the gingival margin. The methods were carried out following all relevant regulations for the use of nonhuman primates following ARRIVE guidelines.

Further, clinical evaluation for ligated sites and gingival tissue samples was obtained at 0.5 (initiation), 1 (early progression), and 3 months (late progression). Determination of periodontal disease at the sampled site was documented by assessment of the presence of BOP and probing pocket depth of >4 mm, as we have described previously (31, 32).

### Tissue sampling and gene expression microarray analysis

A single buccal gingival papillae sample was obtained from either healthy (baseline) or ligature-induced periodontitis-affected tissue from a distinct premolar/molar maxillary region of each animal at each time point using a standard gingivectomy technique (33), and maintained frozen in RNAlater solution. Total RNA was isolated from each gingival tissue using a standard procedure as we have described and tissue RNA samples were submitted to the microarray core to assess RNA quality and analyze the transcriptome using the GeneChip® Rhesus Macaque Genome Array (Affymetrix) (32, 34). Individual samples were evaluated for gene expression analyses. While no specific gene validation was incorporated into this sex-based data analysis we have reported the validation of over 50 of the genes from the complete transcriptome dataset using qPCR analysis (????##).

The expression intensities across the samples were estimated using the Robust Multi-array Average (RMA) algorithm with probe-level quintile normalization, as implemented in the Partek Genomics Suite software version 6.6 (Partek, St. Louis, MO). Differential expression was initially compared using one-way ANOVA across time points within an age group. For genes that had significant mean differences, two sample t-tests were used to investigate differences comparing baseline healthy to disease and resolution samples. Statistical significance was considered by a *p*-value <0.05. Correlation analyses across the genes related to clinical parameters were determined using a Pearson Correlation Coefficient with a *p*-value <0.01. Differences in disease parameters were assessed using a Kruskal–Wallis nonparametric analysis. Gene ontology analysis for biologic processes was conducted using https://reactome.org.

The gene expression data of 18 Young/Adolescent monkeys (9 females, 9 males) were analyzed using BioVinci software (BioTuring Inc), to obtain the graph coordinates for the Uniform Manifold Approximation and Projection (UMAP) and to generate the heatmap of the cluster analyses. The UMAP was generated using the criteria: Data preprocessing None, decision tree criterion Gini, and metric Euclidean. The gene expression data were analyzed using BioVinci software (BioTuring Inc), to obtain the graph coordinates for the Uniform Manifold Approximation and Projection (UMAP) and to generate the heatmaps of the cluster analyses. To create the heatmap, gene expression data were normalized with the lowest value set at 0.00221 and the highest value at 0.0841. The data have been uploaded into GEO accession GSE180588 (https://www.ncbi.nlm.nih.gov/gds).

## Results

### Sex effects on clinical features in younger animals

**Figure 1:**
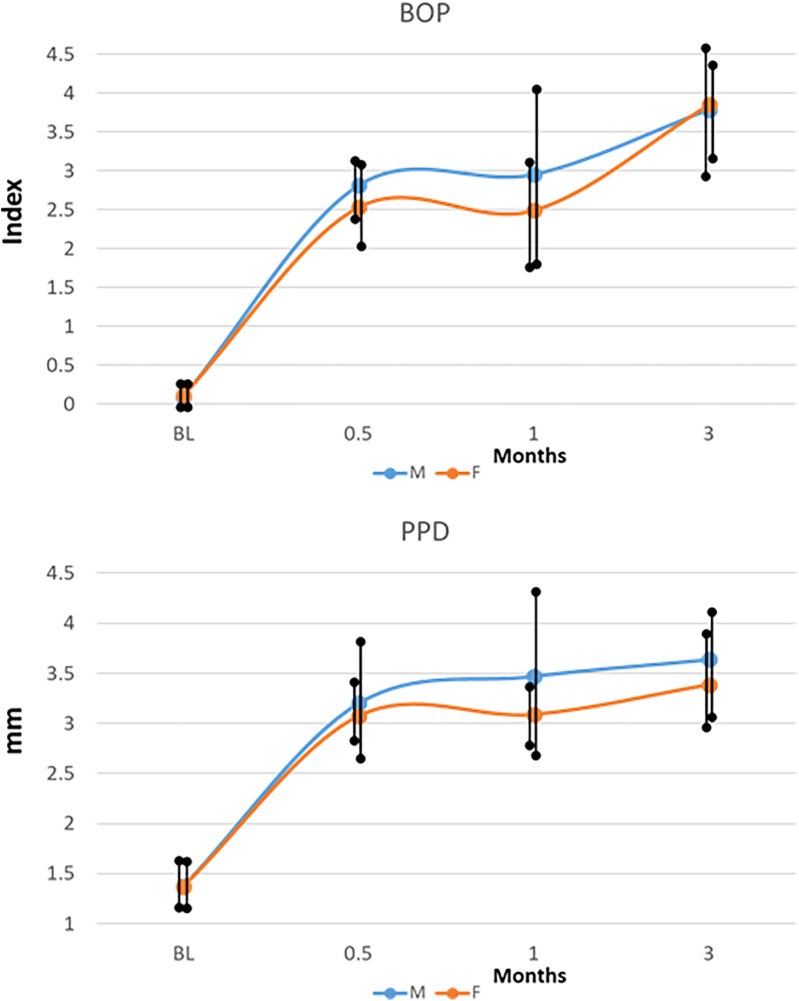
Clinical features (BOP, bleeding on probing; PPD, probing pocket depth) of disease in male **(M)** and female **(F)** animals resulting from ligature placement. The points denote group means for the measures, and the vertical line is 1SD.

Figure 1 summarizes the clinical measures of inflammation and tissue-destructive processes that occurred in the younger animals and varied based on the animal's sex. As we had noted previously (35) there was a significant increase in inflammation (BOP) and probing pocket depth (PPD) in both sexes that occurred as early as 2 weeks (0.5 months) and generally continued to increase through 1–3 months during progressing disease. The male animals showed greater group mean inflammatory responses via BOP at 0.5–1-month disease time points and somewhat greater PPD levels at 1 and 3 months. While these clinical differences did not reach statistical significance, the focus of the study was to investigate the existence of biological differences in the gingival mucosal tissues that may be expressed early in life and could presage altered risk for disease later in life.

### Gingival transcriptomic patterns in health, disease initiation, progression and resolution stratified on sex

The volcano plots (Supplementary Figure S1) show differentially expressed genes (DEG) in the male and female gingival tissues within healthy baseline samples and differential expression at 2-fold or greater (only 4 genes for each sex). Those genes with significant differences (*p* < 0.01) are also identified with 66 elevated levels in females and 48 in male animals. Thus, in healthy gingival tissues from younger animals, a rather limited number of gene differences were seen, albeit patterns of expression related to the sex of the animal could be seen. The distribution of DEGs with disease initiation (0.5 mo.) and progression (1–3 mo.) between the sexes showed that generally, female animals exhibited a greater number of significantly elevated genes than male animals at each time point. In contrast, the male animals demonstrated a larger number of genes that were elevated by 2-fold or greater at all of these time points.

**Figure 2:**
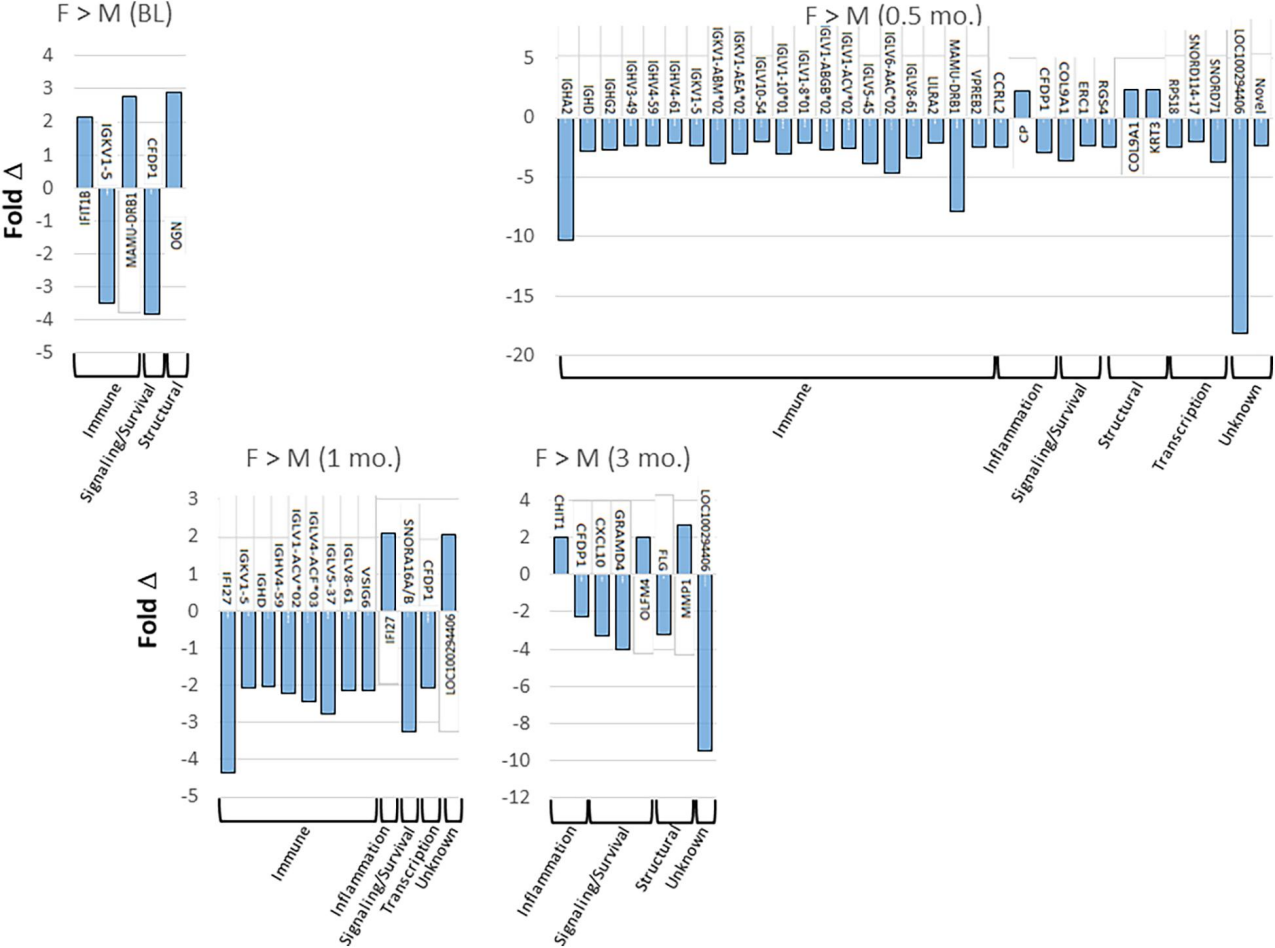
Tabulation of individual genes that were upregulated (positive) or down-regulated (negative) by 2-fold or greater in females and males comparing health (baseline; BL), disease initiation (0.5 mo.), early progression (1 mo.), and late progression (3 mo.).

Figure 2 provides more details on the specific genes that were affected by 2-fold or greater between the sexes in the tissues from health, disease, and resolution. The results for annotated genes demonstrated no specific pattern of elevated genes in healthy tissues. However, with disease initiation (0.5 mo.), a substantial increase in the number of elevated immune response genes was identified in the male animals. With progressing disease immune response genes remained somewhat elevated in the male animals through early progression (1 mo.). In late progression samples, there was no specific profile of altered genes.

**Figure 3:**
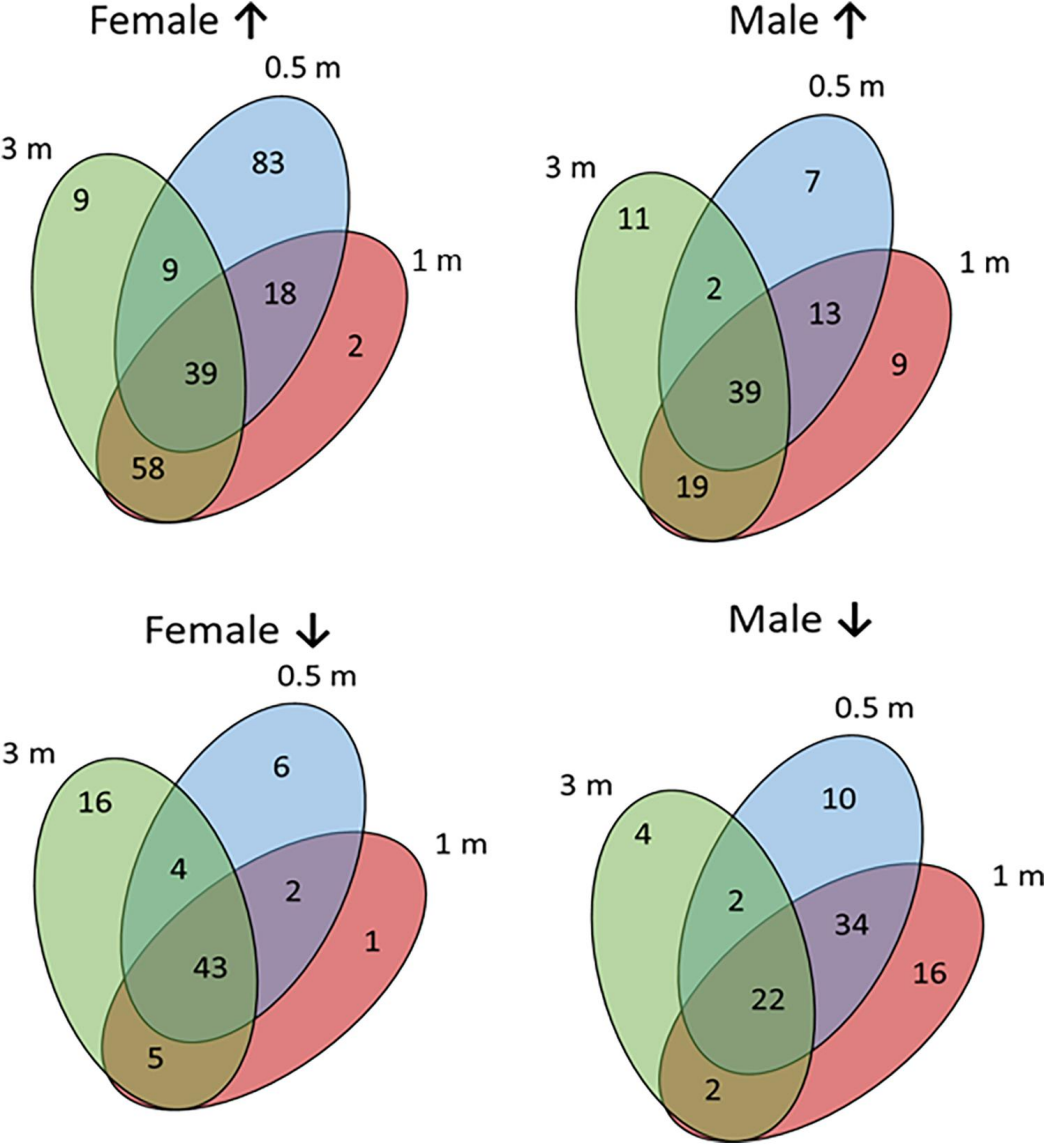
Venn diagrams of specific gene relationships during disease in female and male samples. Numbers of up-regulated (↑) and down-regulated (↓) genes are identified.

Figure 3 presents an approach to identify patterns of specific genes in the sexes highlighting the overlap and unique up-regulated gene profiles in female and male samples compared to baseline healthy tissue levels. The results showed that in females 103 genes are up-regulated by ≥2-fold with disease initiation (0.5 m) and early progression (1 m). A different set of 39 genes were up-regulated across the disease time points (0.5, 1, 3 m). In contrast, there were a more limited number of genes (*n* = 29) showing these types of patterns in male animals, albeit 39 genes were also up-regulated in common throughout the disease process. A similar summarization of the profiles of down-regulated genes vs. baseline samples. In females, the dominant overlapping genes were during all disease time points (*n* = 43). This contrasted somewhat in the male animals with the majority of down-regulated genes (*n* = 60) with initiation and early progression samples.

**Figure 4:**
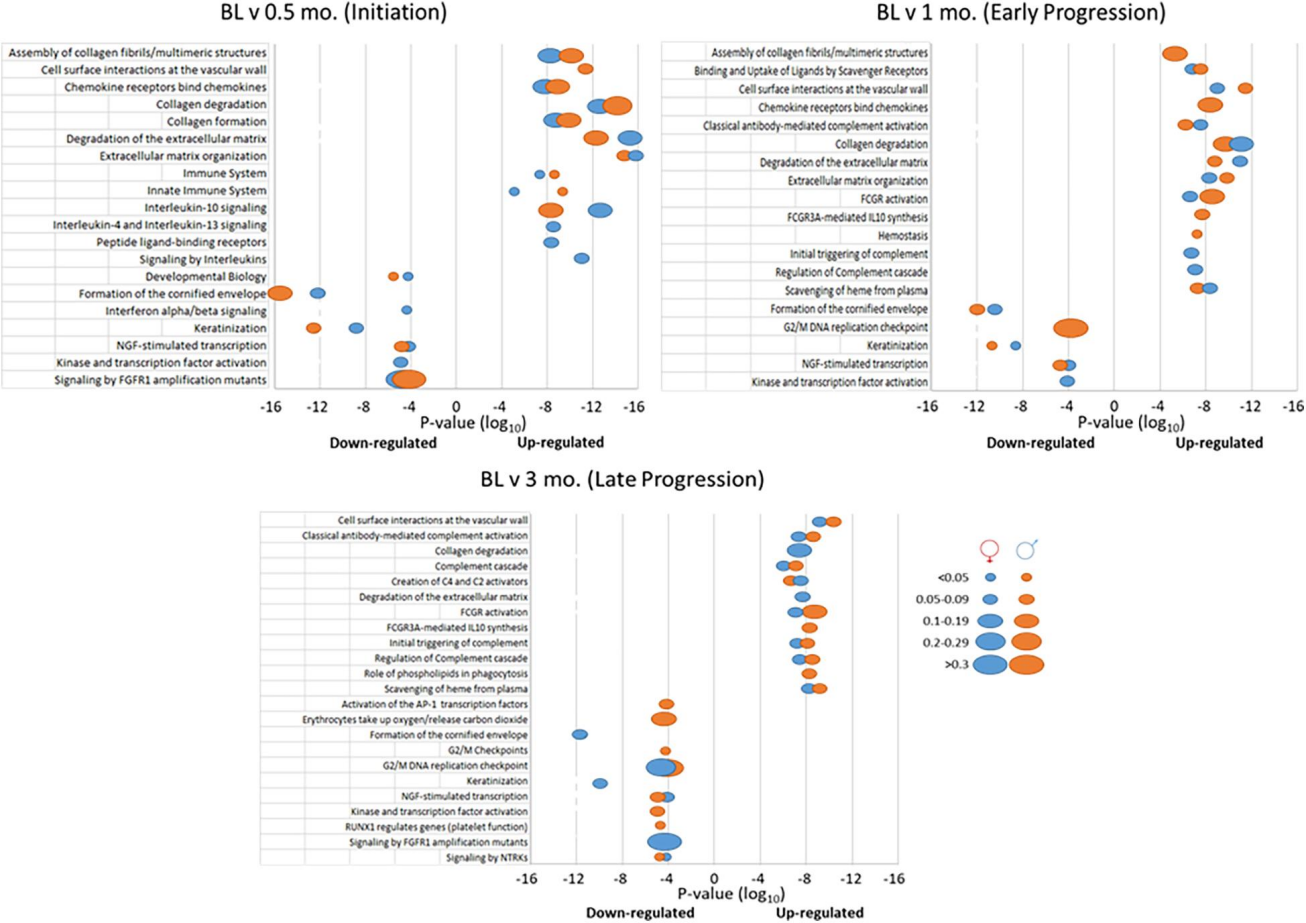
Top 20 biological pathways with overexpressed genes in disease initiation, early progression, or late progression samples compared to baseline (health). *P*-values of the significance of the change are displayed by the ordinate, and the size of the ovals represent the frequency of genes.

Figure 4 summarizes the top 20 biologic pathways that were overrepresented in female or male samples at each of the time points in the study. At disease initiation, an array of host response processes and various tissue integrity and matrix processes were up-regulated compared to baseline samples. However, in this early disease phase, the biology of epithelial cells and cellular transcription processes were down-regulated. While many of these overlapped between the sexes, there were unique features of the altered gene profiles particularly in the female animals. With early lesion progression, the up-regulated processes continued to emphasize a role in tissue integrity and a range of inflammatory response components as the disease progressed, albeit a number of these processes were sex specific. Epithelial cell biological processes also continued to be down-regulated in both sexes. The late progression samples demonstrated nearly total overlap of up-regulated processes between the sexes and maintained the patterns of tissue integrity and host response processes as in early progression. This contrasted somewhat to the down-regulated process findings, whereby the most represented biological processes were often different between the sexes. In this case females showed the greatest impact on epithelial cell functions, with the males were skewed towards transcription and general cellular biology processes. Table 1 provides a summary of the biological pathways reflecting the gene expression differences between female and male samples in healthy and diseased tissues.

**Table 1:** Gene ontology analysis of overexpressed biological pathways in female vs. male samples. Genes included were significantly different at *p* < 0.01.

GO biological process complete	Fold Enrichment	Raw *P* value
Baseline (Health)		
Wound healing, spreading of cells	39.8	8.33 × 10^−5^
Positive regulation of apoptotic process	6.56	3.38 × 10^−5^
Sexual reproduction	4.98	8.61 × 10^−5^
Chromosome organization	4.74	2.11 × 10^−5^
Response to oxygen-containing compound	3.91	5.43 × 10^−5^
Regulation of cell differentiation	3.88	6.03 × 10^−6^
Regulation of apoptotic process	3.7	4.5 × 10^−5^
Regulation of developmental process	3.08	7.92 × 10^−6^
Cellular component assembly	2.78	9.81 × 10^−5^
Cell differentiation	2.46	1.13 × 10^−4^
Initiation (0.5 months)		
Antimicrobial humoral response	43.18	1.0 × 10^−3^
Regulation of cell-matrix adhesion	39.16	1.21 × 10^−3^
Leukocyte chemotaxis	31.48	1.85 × 10^−3^
Negative regulation of cell migration	24.4	2.31 × 10^−4^
Negative regulation of response to external stimulus	18.04	5.54 × 10^−4^
Leukocyte migration	17.82	5.54 × 10^−3^
Chemotaxis	12.54	1.57 × 10^−3^
Defense response	9.28	1.2 × 10^−4^
Positive regulation of cell differentiation	8.13	5.32 × 10^−3^
Response to external stimulus	5.24	1.67 × 10^−3^
Early Progression (1 month)		
Protein deglycosylation	42.45	8.2 × 10^−5^
Cytoplasmic translation	14.84	3.14 × 10^−5^
Apoptotic process	4.51	4.1 × 10^−5^
Protein catabolic process	3.8	9.03 × 10^−5^
Cellular localization	2.5	2.63 × 10^−5^
Transport	2.3	6.64 × 10^−7^
Cellular nitrogen compound metabolic process	2.08	6.92 × 10^−5^
Multicellular organism development	2.06	4.0 × 10^−5^
Developmental process	1.85	9.09 × 10^−5^
Cellular component organization or biogenesis	1.82	4.2 × 10^−5^
Late Progression (3 months)		
Nuclear membrane reassembly	48.11	5.66 × 10^−5^
Cellular extravasation	24.53	3.19 × 10^−5^
Endomembrane system organization	5.62	1.45 × 10^−5^
Vasculature development	4.99	9.62 × 10^−5^
Membrane organization	4.27	6.25 × 10^−5^
Response to nitrogen compound	4.11	4.05 × 10^−5^
Cell cycle	3.63	6.75 × 10^−5^
Cytoskeleton organization	3.42	1.82 × 10^−5^
Regulation of cell population proliferation	3.07	3.77 × 10^−5^
Cellular component organization or biogenesis	2.21	2.63 × 10^−8^

Supplementary Figure S2 provides summary Venn diagram plots of gene expression differences in the sexes during experimental periodontitis. Generally, the profiles at all times points for genes elevated compared to baseline healthy samples showed an extensive overlap in the specific genes in both sexes. At initiation and early progression, the male animals did show 3–4 times the number of uniquely elevated genes compared to female samples; however, the number of these gene differences decreased between the sexes by late progression, with specific increases in females. Fewer genes were down-regulated in both sexes with a dominant overlap at all disease timepoints. However, the sex differences in the number of uniquely down-regulated genes was actually increased in female animals in late progression samples.

**Figure 5:**
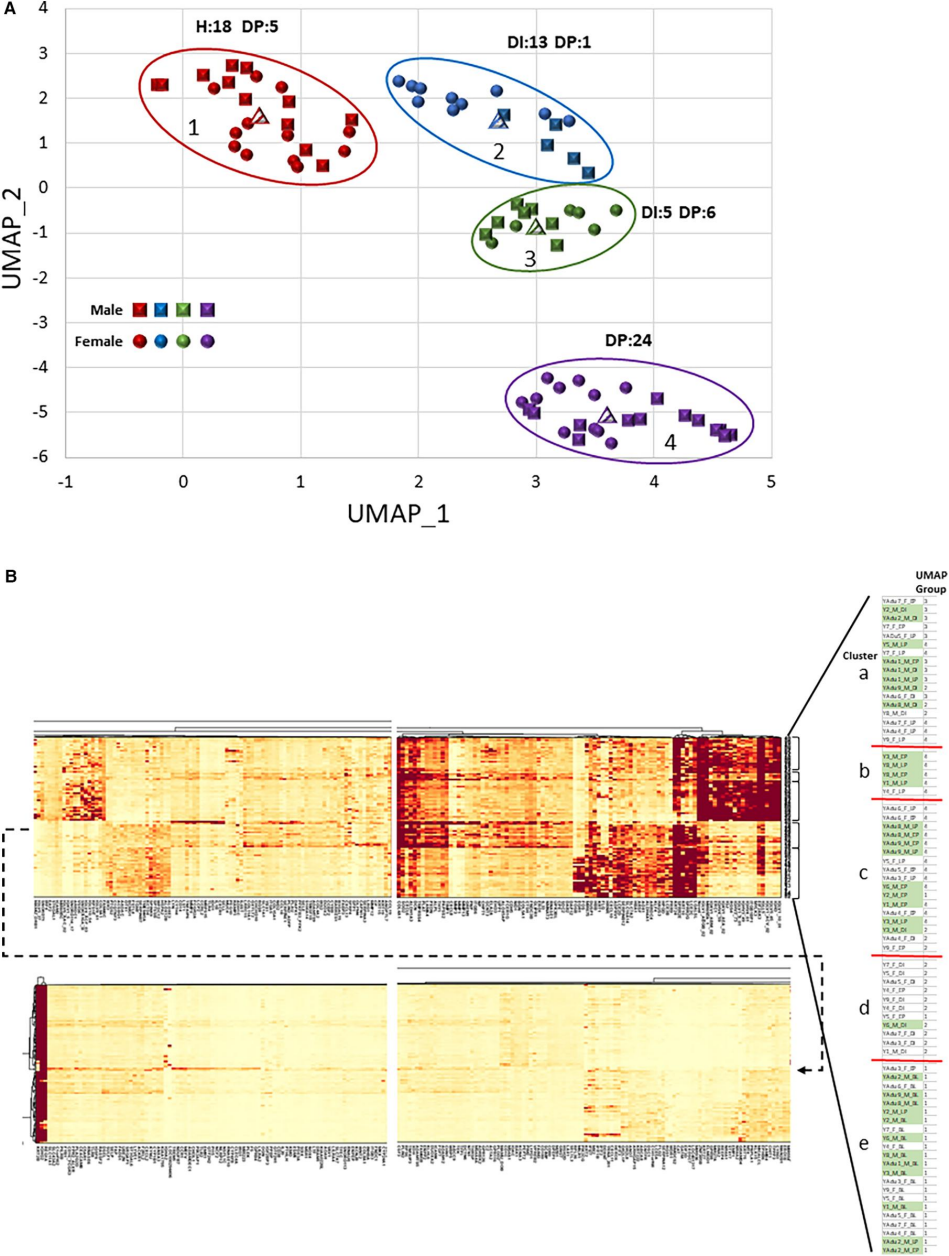
**(A)** UMAP visualization of the clustering of individual gingival tissue samples based upon the transcriptomic expression profiles. Each point denotes the composite expression of an individual animal with squares for males and circles for females. Different colored points denote grouping of samples based upon this composite gene score. The encircled groups also identify the number of H (baseline health), DI (disease initiation), or DP (disease progression) gingival tissue samples in each group. **(B)** Heatmap of cluster analysis based upon individual gene expression (*x*-axis) and organized into 5 clusters of individual samples (*y*-axis), identified on sex, age group, and experimental time point. The blowup table shows the organization of the individual samples with accompanying legend identifying the sample features of each cluster, as well as the UMAP group for each sample.

Based upon the DEGs that were identified in gingival tissues of different sexes in health and disease initiation/progression, a multivariate analysis was performed to identify overall gene expression relationships and clustering of samples stratified by sex, age, and time point within the experimental periodontitis model. Figure 5 depicts the findings from a UMAP and a cluster analysis of these data. First, there were clear differences primarily related to the stage of the disease process, with a mix of sexes in each of the UMAP groups based on the gene expression profiles (Figure 5A). Group 1 was enriched for all healthy samples from both sexes. Group 2 was skewed towards progressing disease in the female animals. Group 4 encompassed exclusively disease progression samples with a similar distribution of sexes. Finally, group 3 included both disease initiation/progression samples, with some skewing towards male samples.

The cluster analysis provided additional details of characteristics of individual gene expression within these profiles across the study population (Figure 5B). Compared to our previous results in adult and aged animals (36), a major cluster discriminator was not the sex of the animals with these gene profiles. Group “a” represented both sexes, with most disease initiation samples in this cluster. The exceptions were the small Group “b”, which was dominated by male samples (80%) and disease tissues, and Group “d”, which was primarily female (91%) and a majority of disease initiation samples. Of interest was that all the baseline (BL) healthy samples were in Group “e”. Finally, generally, the heatmap clusters and the UMAP groups were consistent. Thus, this comparison of transcriptomes demonstrated that the features and kinetics of lesion formation appeared to be the dominant features, vs. the sex or age within this young cohort of animals, in driving gene expression differences in the gingival tissues.

### Gingival transcriptomic patterns related to clinical features of disease: sex differences

Supplementary Figure S3A summarizes the relationship between the gingival gene transcriptome and the clinical disease features of bleeding on probing (BOP) and probing pocket depth (PPD) during ligature-induced disease. The frequency of total sample positive and negative gene correlations unique to BOP was greater in males. In contrast, the unique gene correlations with PPD were elevated in the females, as well as the number of genes that showed overlapping correlations with both BOP and PPD. In males, the BOP/PPD overlapping genes were decreased compared to unique gene correlations with either clinical measure. In healthy and resolution samples there was an overall lower prevalence of any correlations with BOP or PPD in both sexes, except positive correlations with BOP in the female animals. This contrasted with the features in the disease sample, whereby in both sexes there were a much greater number of significant correlations of these genes with the PPD measures, and nearly all of these were unique relationships to only the PPD levels.

Supplementary Figure S3B summarizes the distribution and relationship of correlations of altered gene expression between the sexes for both BOP and PPD. Noted features included similar unique numbers of correlated total genes in both sexes with BOP, albeit numerous overlapping genes between the sexes. This contrasted with the features of PPD, whereby unique correlated genes, both positive and negative, dominated in the female samples, with overlapping genes primarily relegated to negative correlations. The sex differences were also highlighted in stratifying the correlations based on health/resolution or disease samples. Correlations in health/resolution were skewed towards the female sample related to BOP, with little differences in sex associated with PPD measures. In contrast, in disease samples, the primary correlations were observed in female samples with the PPD values.

Supplementary Figures 4A,B provides details on the specific genes that comprised these correlations. Positive correlations with BOP in health in both sexes showed similar gene profiles that included an array of cytokines, chemokines, immune cell markers, and adaptive immune components (291–330). In disease samples, negative correlations were seen in both sexes in tissue structural genes with BOP. However, specifically in the females, positive correlations were noted with 171–190 (adaptive immune responses) with BOP.

Similar analytics for gene expression and PPD are displayed in the figure. A striking feature was the distribution of correlated genes that was distinctive between the sexes. In healthy tissues in males, 41–60, 161–190, 291–300, 321–330 were positive, while frequently negatively correlated in females. These associations represented biological functions of inflammation, adaptive/innate immune responses, and transcription-related genes. In contrast, genes 211–220 were highly positively correlated in healthy tissues from only females, representing mostly structural genes in the gingival tissues. With disease, a more limited repertoire of correlations was observed, particularly 161–190 were positive only in females.

## Discussion

This study utilized a human-like model of experimental periodontitis in nonhuman primates to explore the gingival molecular aspects of periodontal lesion initiation and progression in young individuals stratified by sex. This model provides a prospective capacity to examine the dynamics of lesion development concomitant with changes in juxtaposed tissue host responses. Nonhuman primates provide a clear preclinical model for examining an array of human diseases (22). Related to oral disease, and in particular periodontal disease, clinical, microbiological, and immunological characteristics of periodontal lesions (21, 30) that are modulated by age, sex, obesity, and heritability (26–28, 37) reflect the human conditions. Recently, we reviewed the inherent value of studies in macaque monkeys for modeling human periodontal disease (23). Using this model we have demonstrated an array of local transcriptomic disparities and systemic inflammatory/immune response alterations that were related to age across the lifespan (32), as well as based upon sex (24, 25), and demonstrated features of these responses with disease expression related to sex (25, 35, 36).

Existing data provides strong evidence for the increasing importance of considering sex as a biological variable related to disease risk, diagnosis, and therapeutic outcomes (38, 39) in cancer, cardiovascular diseases, infectious, autoimmune, and chronic inflammatory diseases (40–44). Periodontitis has been reported to be modified via a variety of demographic/behavioral factors, including a greater prevalence in males (15, 45), strongly modulated with age (11, 46–48), and significantly affected by obesity (49, 50), diabetes (51–54), stress (10, 55), and smoking (16). Nevertheless, a complicating facet of this disease is that in humans, generally the onset of the disease is not identified until after about 35 years of age (46), while accepted risk factors including oral microbiome pathogens (56, 57) and genetic risk determinants (58) are present early in life. These reports continue to emphasize the importance of experimental designs for biological disease studies that diligently incorporate sex is a major factor in group stratification, management/treatment, and analysis. Understanding the ramifications of these features is difficult to address with human disease, particularly if longer term risk is displayed by sex differences in gingival mucosal responses that can be observed in young individuals. Thus, we evaluated the gingival transcriptome of the disease process in nonhuman primates who approximated 10–25 years old humans. The hypothesis tested was that early in life, response programming of mucosal tissue, such as the gingiva would present sex-based alteration in differentially expressed genes related to periodontal biological processes.

Clear patterns of sex-related responses were noted in healthy tissues that did not differentially increase in magnitude during disease in these younger animal samples. However, generally, male animals showed a greater frequency of elevated gene expression during disease compared to female animals. This differed from similar results in adult and aged animals whereby during disease, gene expression patterns showed greater similarity between the sexes (36). Nevertheless, the gene profiles showed distinct differences between the sexes related to those gene identifiers demonstrating the greatest magnitude of change. This was particularly noted in adaptive immune genes in males, particularly with disease initiation and early progression. Both up-regulated and down-regulated genes in disease or resolution compared to baseline healthy tissue expression showed considerable overlap between the sexes. While each sex did demonstrate some uniquely altered genes, generally 50%–70% of the altered genes were common between the sexes. A distinct observation in this sex-based comparison was a limited number of genes that demonstrated a multi-fold difference between the sexes at any of the time points. The majority of the dissimilarities were smaller, but significantly increased or decreased between the sexes.

The gene expression profile differences with sex or disease were highlighted by identifying that clusters or groups of samples appeared to be primarily based upon the disease or health status of the tissues within this experimental model, with less difference distinguished by the sex in these younger animals. Overrepresented biologic pathways of sex-based gene expression during disease initiation, progression, and resolution provided discrete similarities between the sexes. While the top 20 pathways differed with different stages of the disease process, the majority of the highlighted biology was similar in males and females. Also, this was characterized by the direction of change, either up- or down-regulated, which was identical between the sexes.

Human data and more limited results from nonhuman primate studies establish the presence and alterations in local adaptive immune reactions (59, 60) as vital components in the initiation and progression of periodontal lesions. In these younger animals, a rather limited repertoire of genes showed sex-related differences in healthy tissues, with no specific biological patterns. Altered biological pathways of innate immune and inflammatory responses help describe the chronic immunoinflammatory lesions of periodontitis (61, 62). The substantial increases in males, in the number of adaptive immune response genes with disease initiation and early progression, may reflect overall differences in the balance of inflammatory and immune response attributes in male vs. female animals. While sex hormones have been identified to affect host responses to exogenous or endogenous challenges (41, 42, 63, 64) for systemic conditions, their impact on the structure/functions of the gingival mucosal tissues remains unclear.

The robust dataset derived from bulk transcriptomic analytics includes biological information on an array of cell types/functions within these tissues. When integrated into an analysis to identify gene expression patterns related to sex, age, and diseased-based samples, in this younger group of animals, the driving influence appeared to be primarily focused on the sampling time point of the experimental periodontitis model. Thus, within the UMAP groups or clusters, sex and/or age of the sample showed less discriminating power than clinical health or disease of the tissues. This differed from patterns in older animals whereby sex was an important distinguishing facet in the patterns of gene expression (36).

The gene expression results were also evaluated concerning the profiles of clinical changes in bleeding on probing (BOP; inflammation) and probing pocket depth (PPD; tissue destruction). In this case, females showed a greater relationship between altered gene expression and PPD, while males showed a skewed relationship of gene expression with BOP. Interestingly, examination of healthy samples showed females with a large array of genes positively correlated with BOP levels. While BOP is not necessarily a good predictor of progression to periodontitis (65), this finding could support that younger females may mount a more rapid immunoinflammatory response to control the microbial insult, which may be lost with aging/menopause increasing disease risk. In contrast, in the disease samples, the dominant gene correlations in both sexes were with PPD values. Finally, we observed extensive correlations of gene expression levels with BOP that were unique and overlapping in both males and females. A similar assessment with PPD clinical features demonstrated a skewing toward the correlations in the female animals. Thus, while these findings may a bit confusing regarding the observations in humans of greater disease in males, this could reflect a more rapid/robust immunoinflammatory response in females that actually can better control the disease progression. Moreover, cross sectional human population studies do not accurately define the actual dynamic phase of the clinical measures of the disease process. Moreover, studies using human gingival tissues have revealed significantly varied profiles of differential gene expression in periodontitis vs. healthy tissues (66); however, definitive relationships of the transcriptome components to clinical measures of periodontitis were not provided, nor was stratification of the differences based on sex described. Our model provides new insights into existing biological variations reflected by the gene expression profiles that may contribute to clinical differences that are noted between sexes. The findings also provide an interesting perspective on the clinical-molecular linkage between inflammation and tissue destruction. More specifically, reports in humans (46), and our findings in nonhuman primates (25, 26) indicates a more destructive inflammatory disease process in males. The response differences in this report were limited to transcriptomic features of host tissues that showed sex effects on both immune/inflammatory and structural components of these mucosal tissues. For more robust translational interpretations, further studies would benefit by assessing specifically targeted biomolecules within this changing disease environment to more fully document sex differences.

While the nonhuman primate model of experimental periodontitis provides a level of control over the disease process, not feasible in humans, limitations of the study include that this clinically similar example of oral disease initiation and progression may have biological processes that are different from longer-term naturally occurring disease in humans. Nevertheless, it would be expected that sex-related transcriptome differences could be observed in human gingival tissues. Additionally, a caveat is that this study only documented the features of the transcriptome in these tissues. While data generally support that message and product changes show some relationship, a comparison with the biomolecular biology in chronic lesions in humans may exhibit some critical differences. Furthermore, challenges with using this large animal preclinical model include ready access to animals with the appropriate oral health parameters to initiate the study, as well as the inherent costs of implementing the study through NIH supported national primate centers. Thus, the inclusion of 18 animals os separate sexes across this prospective longitudinal study limits the overall power of the correlation analyses utilized to explore the relationship of specific host response features with the clinical presentation of the gingival tissues. Finally, we have identified some differences in the autochthonous oral microbiota in younger vs. older nonhuman primates (29). Thus, while there is substantial similarity in the qualitative composition of the microbiome in nonhuman primates and humans, the interaction of the quantitative traits of the microbiomes with the juxtaposed oral mucosa may result in diverse response profiles.

This model of the formation and resolution of periodontal lesions provides insights into the dynamics of transcriptomic responses in gingival tissues related to sex. While in these younger animal samples, the time points of health or disease in the model provided the major driver for transcriptome variations, some sex differences were detected even at an early age. A conundrum in the field is the apparent existence of risk factors for periodontitis early in life (e.g., genetics, microbiome, sex), albeit the disease is generally not expressed until the 4th decade and later in life of humans. Potentially these early differences could help to identify markers of risk for expressing periodontitis based upon sex differences.

## Data Availability

The datasets presented in this study can be found in online repositories. The names of the repository/repositories and accession number(s) can be found in the article/Supplementary Material.

## Supplementary material

Supplementary Figure S1

Volcano plots of differentially expressed genes (DEGs) in female and male samples in healthy (baseline) gingival tissues and gingival tissue samples during disease (0.5, 1, 3 mo.). Red dots denote gene expression that was elevated in females, and purple dots denote gene expression levels increased in males. The blue rectangles enclose genes that were elevated in one sex at 2-fold or greater. The green rectangles enclose genes that were significantly different between the sexes at least at *p* < 0.01. Numbers signify the gene frequency in each of the boxes.

Supplementary Figure S2

Venn diagrams of altered gene expression for up-regulated or down-regulated genes compared to baseline (healthy) tissues stratified by sex. Numbers denote genes altered in expression at the different disease [0.5 m (disease initiation - DI, 1 m (early progression - EP, 3 m (late progression, LP)] time points.

Supplementary Figure S3

(**A**) Venn diagrams presenting correlations of gene expression values with clinical measures of inflammation (BOP) and tissue destruction (PPD). Correlations across all time points (Total), in health/resolution samples, and in all diseased samples are presented in the 3 panels. Numbers denote gene correlations present as unique or overlapping between BOP and PPD stratified by sex. (**B**) Venn diagrams presenting correlations of gene expression values with BOP and PPD. Numbers denote gene correlations as unique or overlapping between sexes, stratified by clinical measure.

Supplementary Figure S4

(**A**) Ribbon chart identifying patterns of gene correlations with BOP or PPD clinical measures in baseline healthy (H) or disease (D) tissues. Blue ribbons denote magnitude of positive correlation and red ribbons denote magnitude of negative correlation for the 393 individual genes. Data are stratified by sex, and (**B**) individual gene IDs are included in the table.
