# Supplementary material for: Sex effects on early life features of the gingival transcriptome
Source: Front Dent Med. 2025 Oct 28;6:1653315. doi: 10.3389/fdmed.2025.1653315 (PMC12602430; doi:10.3389/fdmed.2025.1653315)
Supplement: Supplementary file 1 [file Supplementaryfile1.pptx]

## Slide 1
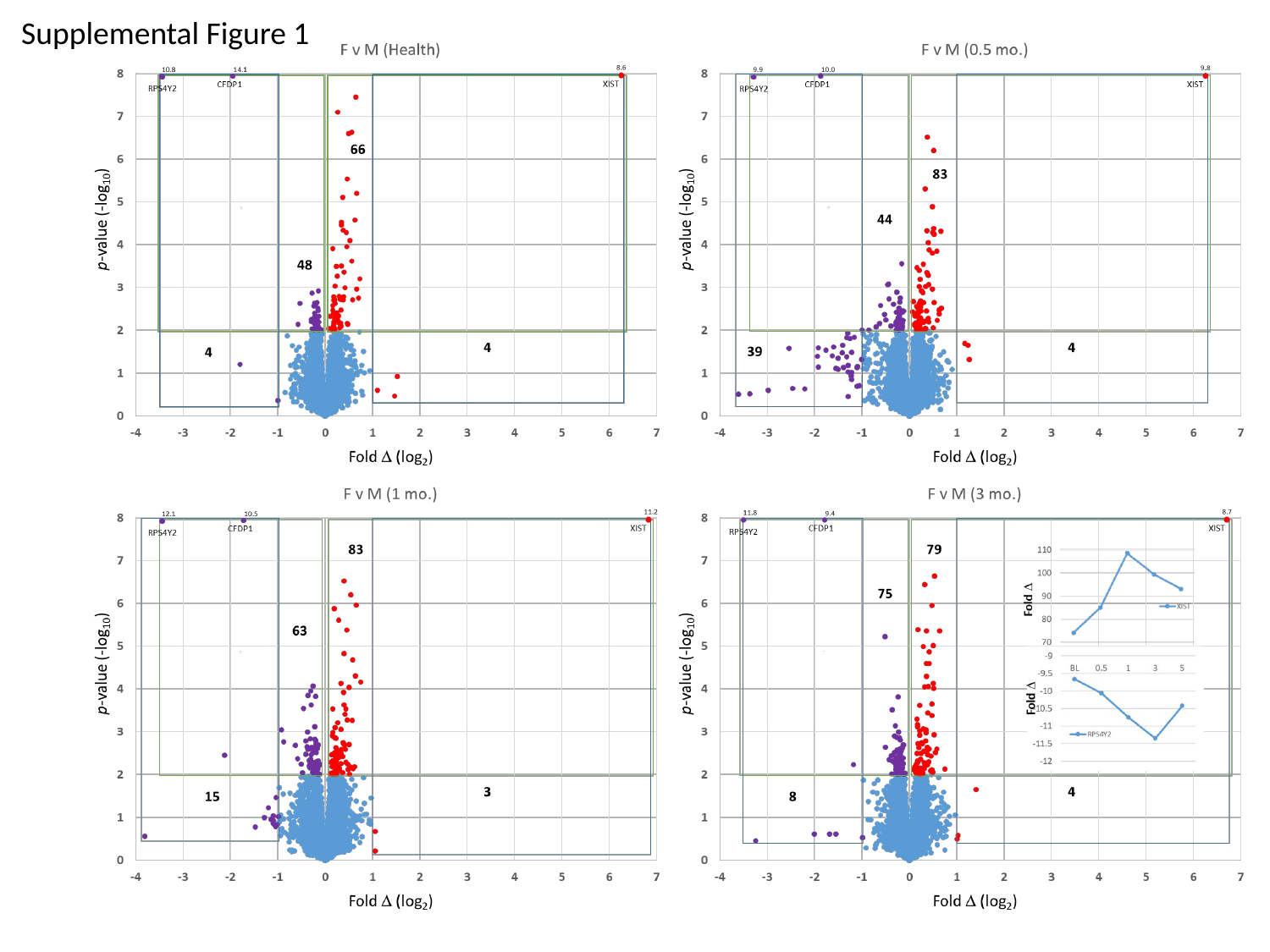

Supplemental Figure 1

## Slide 2
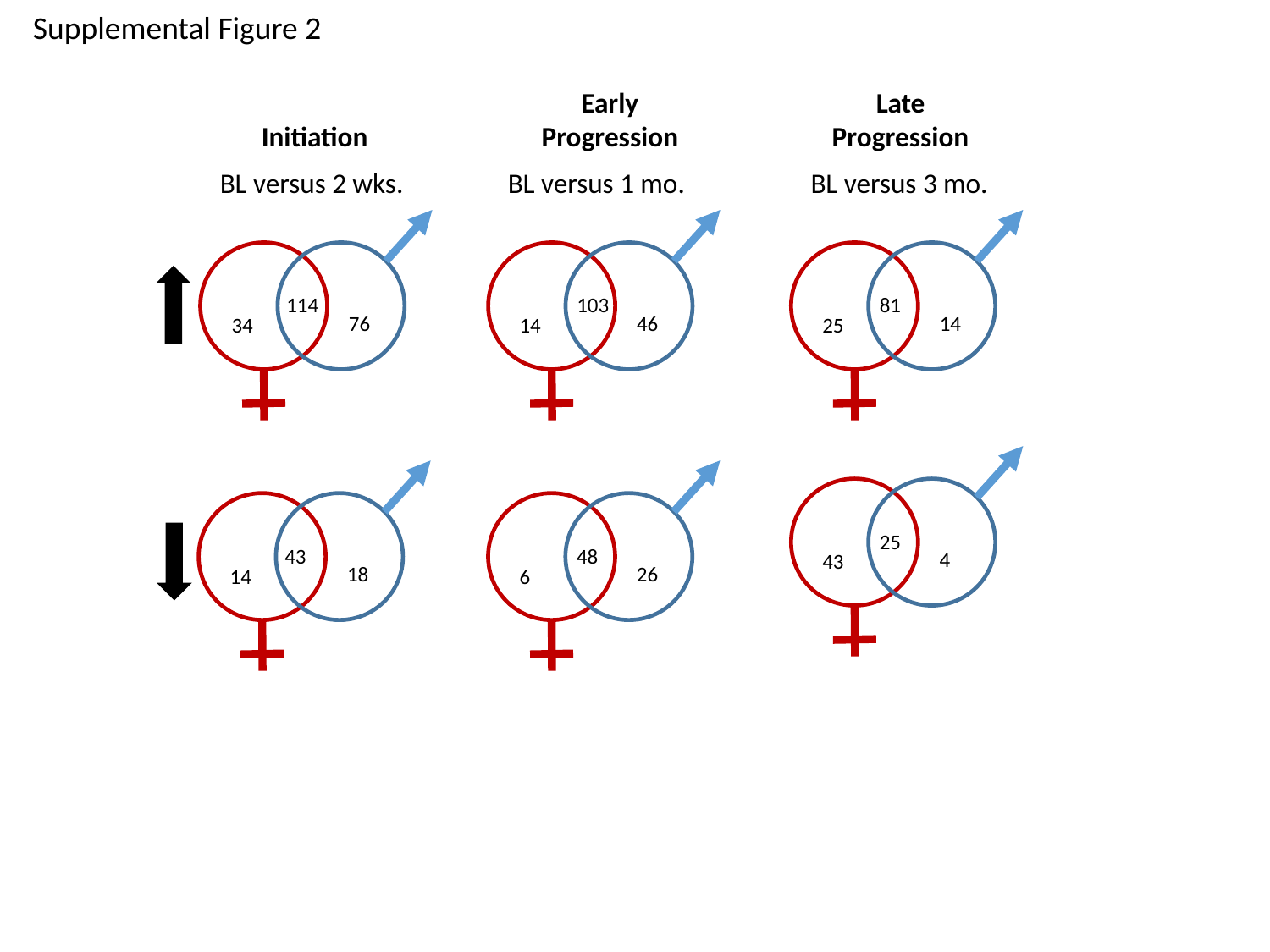

Supplemental Figure 2
Early
Progression
Late
Progression
Initiation
BL versus 2 wks.
BL versus 1 mo.
BL versus 3 mo.
114
103
81
76
46
14
34
14
25
25
43
48
4
43
18
26
14
6

## Slide 3
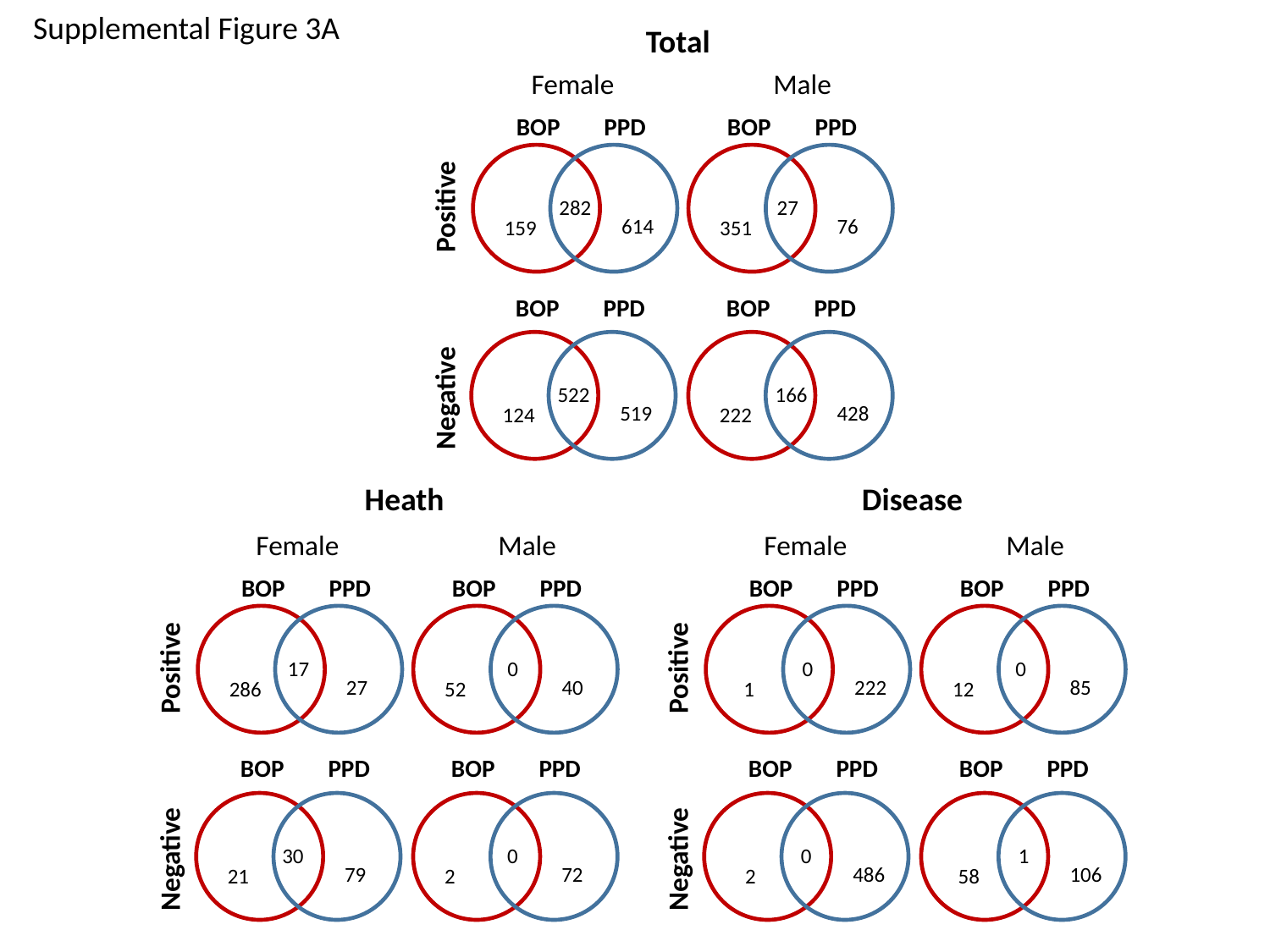

Supplemental Figure 3A
Total
Female
Male
BOP PPD
BOP PPD
Positive
282
27
614
76
159
351
BOP PPD
BOP PPD
Negative
522
166
519
428
124
222
Heath
Disease
Female
Male
Female
Male
BOP PPD
BOP PPD
BOP PPD
BOP PPD
Positive
Positive
17
0
0
0
27
40
222
85
286
52
1
12
BOP PPD
BOP PPD
BOP PPD
BOP PPD
Negative
Negative
30
0
0
1
79
72
486
106
21
2
58
2

## Slide 4
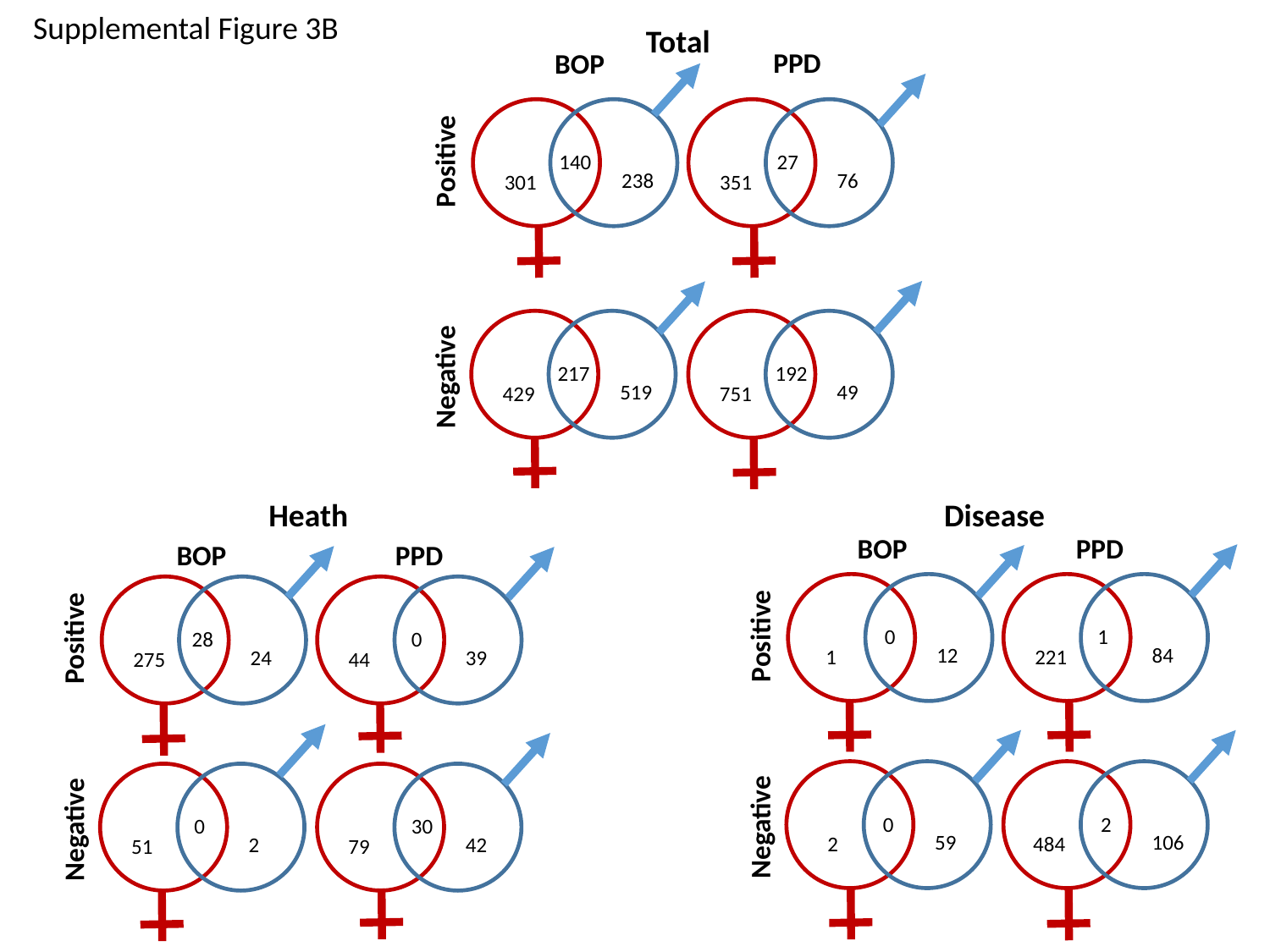

Supplemental Figure 3B
Total
PPD
BOP
Positive
140
27
238
76
301
351
Negative
217
192
519
49
429
751
Heath
Disease
PPD
BOP
PPD
BOP
Positive
Positive
0
1
28
0
12
84
1
221
24
39
275
44
Negative
0
2
Negative
0
30
59
106
484
2
2
42
51
79

## Slide 5
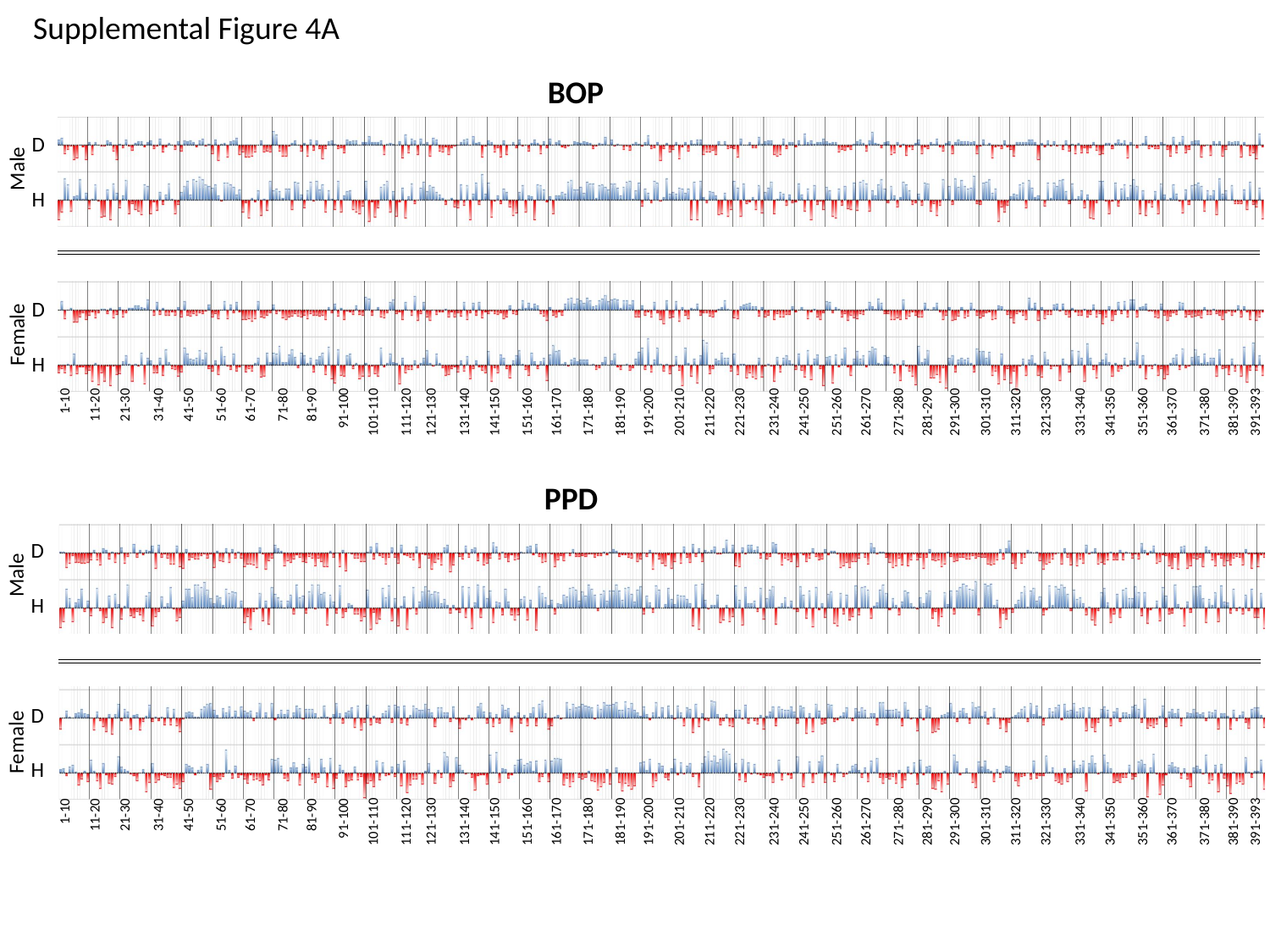

Supplemental Figure 4A
BOP
D
H
D
H
Male
Female
391-393
111-120
291-300
301-310
311-320
321-330
331-340
341-350
351-360
361-370
371-380
381-390
1-10
11-20
21-30
31-40
41-50
51-60
61-70
71-80
81-90
91-100
101-110
121-130
131-140
141-150
151-160
161-170
171-180
181-190
191-200
201-210
211-220
221-230
231-240
241-250
251-260
261-270
271-280
281-290
PPD
D
H
D
H
Male
Female
391-393
111-120
291-300
301-310
311-320
321-330
331-340
341-350
351-360
361-370
371-380
381-390
1-10
11-20
21-30
31-40
41-50
51-60
61-70
71-80
81-90
91-100
101-110
121-130
131-140
141-150
151-160
161-170
171-180
181-190
191-200
201-210
211-220
221-230
231-240
241-250
251-260
261-270
271-280
281-290

## Slide 6
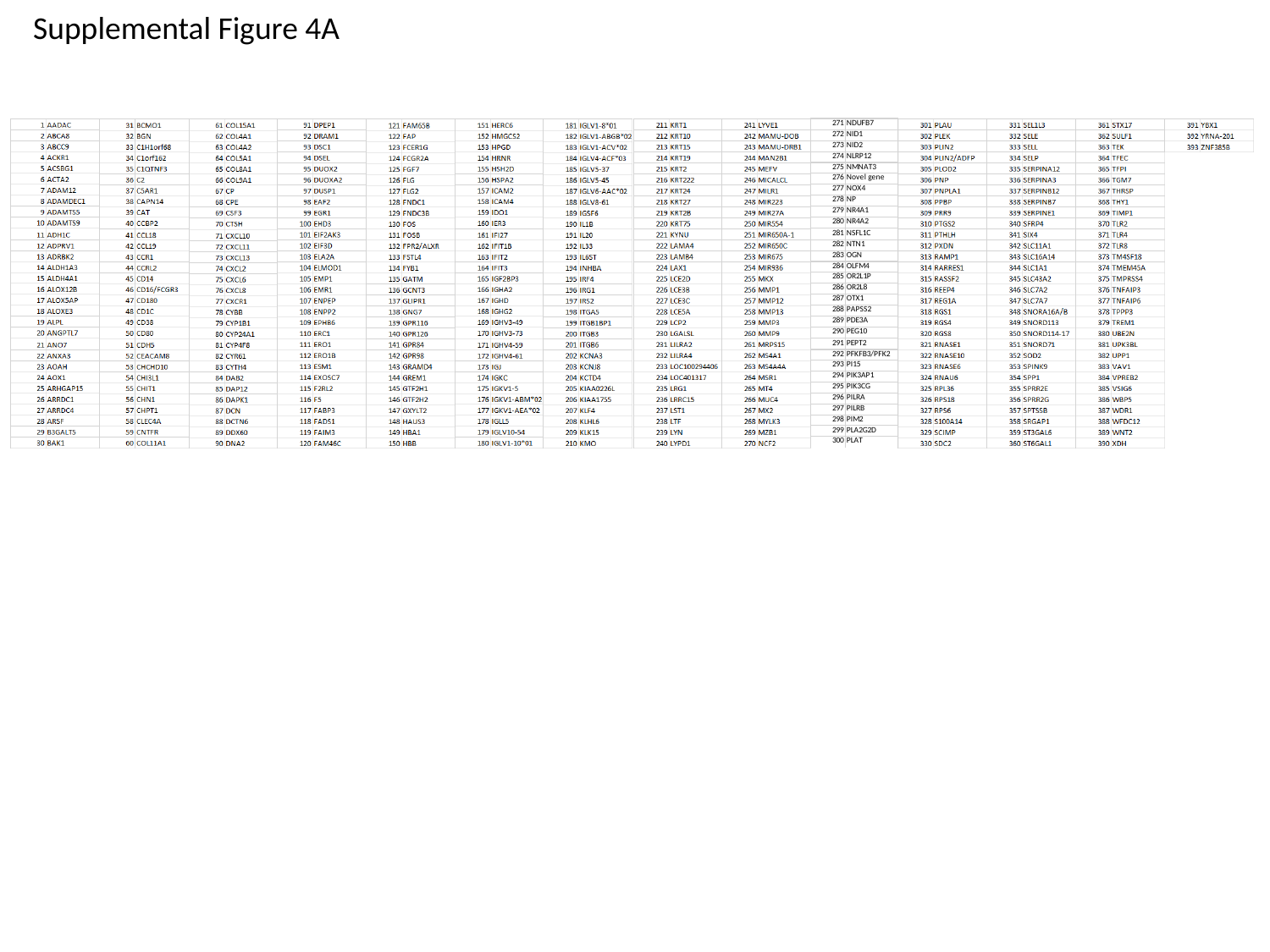

Supplemental Figure 4A
